# Supplementary material for: Quantum non-demolition readout of an electron spin in silicon
Source: Nat Commun. 2020 Mar 2;11:1144. doi: 10.1038/s41467-020-14818-8 (PMC7052195; doi:10.1038/s41467-020-14818-8)
Supplement: Supplementary file 1 — Supplementary Information [file 41467_2020_14818_MOESM1_ESM.pdf]

Quantum non-demolition readout of an electron spin in silicon

Yoneda *et al.*

Supplementary Note 1: Qubit Relaxation

To investigate the mechanism of the observed qubit state relaxation, we perform several control experiments (Supplementary Fig. 1a). In these experiments, we first control the qubit spin by a 10 μs-long microwave frequency-chirped by 10 MHz. After applying different numbers of ancilla measurement pulses, we read out the qubit spin and record the maximum (on-resonance) and minimum (off-resonance) spin-up probabilities, $p_{\uparrow}^{\mathrm{max}}$ and $p_{\uparrow}^{\mathrm{min}}$. Each ancilla measurement is 45 μs long and repeated every 75 μs (= t_{cycle}). Their difference $\delta p_n = p_{\uparrow}^{\mathrm{max}} - p_{\uparrow}^{\mathrm{min}}$ is expected to follow $\delta p_n = \left(\rho^{\downarrow} + \rho^{\uparrow} - 1\right)^n \delta_0$, where δ_0 is a constant that explains the state preparation and measurement errors. From the fit, we obtain the total spin relaxation time T_1^{tot} defined through $\exp\left(-t_{\mathrm{cycle}}/\,T_1^{\mathrm{tot}}\right) = \rho^{\downarrow} + \rho^{\uparrow} - 1$.

In Supplementary Fig. 1b, we plot δp_n along with the fitting results in three different conditions. In all cases, the exchange pulses for a controlled rotation are deactivated. In cases ii and iii, we turn off the reflectometry carrier signals during the ancilla readout process. In case iii, we offset the gate-voltage pulse level at the ancilla measurement stage from the reservoir Fermi level by 5 mV (corresponding to ~ 0.1 meV). T_1^{tot} shows a noticeable improvement from T_1^{tot} = 2.4 ms in the QND readout condition (calculated from $T_1^{\downarrow(\uparrow)}$) only in case iii, which indicates enhanced qubit relaxation during the ancilla readout process, presumably due to cotunneling. Further studies may be necessary to reveal the microscopic mechanisms of the phenomenon.

Supplementary Note 2: Joint Probability Analysis with the Sensor Signal Distribution

The qubit-state dependence of our QND readout performance (F_{M} and F_{P}) is partly explained by qubit-state dependent f_i ($f_i^{\downarrow}$ = 85% and $f_i^{\uparrow}$ = 75%), see Supplementary Fig. 2b. A possible cause of this is ancilla-state dependent fidelity of the ancilla spin readout. The ancilla readout is a two-stage process, as is usually the case with single-shot destructive readout of single electron spins[10]. The first stage correlates the spin state to the presence and absence of tunneling events (spin-to-charge conversion). In the second stage, we detect a blip in the sensor signal as a result of such tunneling events, typically by setting a threshold (charge discrimination). Both stages are prone to errors which, in principle, may be modelled through device and setup parameters. While such detailed characterization is out of the scope of this study, one can anticipate that the charge discrimination infidelity overwhelms the relaxation and thermal broadening effects in our case, given the short measurement time (compared to the relaxation time) and the large Zeeman splitting (with respect to the thermal energy).

To evaluate the effect of charge discrimination errors, we calculate the joint probability $P(m_i m_{30})$ using the histograms of sensor signals, D^{u} and D^{d}, for traces with and without blips,

respectively (see Supplementary Fig. 3a and Supplementary Note 3). f_i extracted in this manner, $\tilde{f}_i$ (rather than using thresholded values as in the main text) would be free from the charge discrimination errors and describe the errors in converting the qubit spin state to an ancilla electron tunnelling event. We obtain these qubit-to-ancilla-blip conversion fidelities as $\tilde{f}_i^{\downarrow}$ = 86% and $\tilde{f}_i^{\uparrow}$ = 82% (Supplementary Fig. 3c). Improvements from f_i are consistent with the charge discrimination fidelities ($f_{\mathrm{th}}^{\mathrm{d}}$ = 98.2% and $f_{\mathrm{th}}^{\mathrm{u}}$ = 90.0% at the threshold of 20.6 mV, see Supplementary Fig. 3b).

We can furthermore obtain the sensor signal histograms given the input spin state, $D^{\downarrow}$ and $D^{\uparrow}$ (see Supplementary Fig. 3d) through $D^{\downarrow} = \tilde{f}_i^{\downarrow} D^{\mathrm{d}} + \left(1-\tilde{f}_i^{\uparrow}\right) D^{\mathrm{u}}$ and similarly for $D^{\uparrow}$. Note that these can be used (in place of $\Theta_{\downarrow,m_i}\left(f_i^{\downarrow}\right)$ and $\Theta_{\uparrow,m_i}\left(f_i^{\uparrow}\right)$) to calculate the likelihoods of initial as well as posterior spin states, however, the fidelity enhancement we observe is only marginal. It is illustrative to see how well approximated the ratio $D^{\downarrow}/D^{\uparrow}$ is by the step-function as used in the main text (see Supplementary Fig. 3d), because of large qubit-to-ancilla-blip conversion infidelities $1-\tilde{f}_i$ compared to charge discrimination errors $1-f_{\mathrm{th}}^{\mathrm{d(u)}}$. We note that the threshold of 20.6 mV is in fact chosen such that $D^{\downarrow} = D^{\uparrow}$ at the threshold.

Supplementary Note 3: Sensor Signal Distribution Estimation

This section describes our procedure to estimate the distribution functions D^{u} and D^{d} of the peak-to-peak charge sensor signal v with and without detectable blips due to tunneling events, respectively. We denote the probability of finding v below the threshold v_{th} by $p_{\mathrm{th}}^{\mathrm{d}}$, and introduce the fidelity $f_{\mathrm{th}}^{\mathrm{u}}$ $\left(f_{\mathrm{th}}^{\mathrm{d}}\right)$ to describe the probability that v is above (below) v_{th} when there are (no) blips. It then follows that $p_{\mathrm{th}}^{\mathrm{d}} = p^{\mathrm{d}} f_{\mathrm{th}}^{\mathrm{d}} + \left(1-p^{\mathrm{d}}\right)\left(1-f_{\mathrm{th}}^{\mathrm{u}}\right)$, where p^{d} is the probability of traces without blips. Using these notations, the sensor-signal distribution $D\left(v, p_{\mathrm{th}}^{\mathrm{d}}\right)$ can be expressed as

$$D\left(v, p_{\mathrm{th}}^{\mathrm{d}}\right) = p^{\mathrm{d}} D^{\mathrm{d}}(v) + \left(1-p^{\mathrm{d}}\right) D^{\mathrm{u}}(v) = A(v) p_{\mathrm{th}}^{\mathrm{d}} - B(v), \quad (1)$$

where $A(v) = \frac{D^{\mathrm{d}}(v) - D^{\mathrm{u}}(v)}{f_{\mathrm{th}}^{\mathrm{d}} + f_{\mathrm{th}}^{\mathrm{u}} - 1}$ and $B(v) = \frac{\left(1-f_{\mathrm{th}}^{\mathrm{u}}\right) D^{\mathrm{d}}(v) - f_{\mathrm{th}}^{\mathrm{d}} D^{\mathrm{u}}(v)}{f_{\mathrm{th}}^{\mathrm{d}} + f_{\mathrm{th}}^{\mathrm{u}} - 1}$. We can extract parameters $A(v), B(v)$ for each v bin (with a bin width of 0.5 mV in our case) by fitting $D\left(v, p_{\mathrm{th}}^{\mathrm{d}}\right)$ as a function of $p_{\mathrm{th}}^{\mathrm{d}}$. When $D^{\mathrm{u}}(v) \gg D^{\mathrm{d}}(v)$, $B(v)/A(v) = f_{\mathrm{th}}^{\mathrm{d}}$. From the average values of $B(v)/A(v)$ for 28 mV $\leq v \leq$ 36 mV, we obtain $f_{\mathrm{th}}^{\mathrm{d}}$ (98.2% for v_{th} = 20.6 mV). In the opposite case when $D^{\mathrm{d}}(v) \gg D^{\mathrm{u}}(v)$, $B(v)/A(v) = 1 - f_{\mathrm{th}}^{\mathrm{u}}$. The average of $1 - B(v)/A(v)$ in the range of 9 mV $\leq v \leq$ 11 mV yields $f_{\mathrm{th}}^{\mathrm{u}}$ (90.0% for v_{th} = 20.6 mV). Finally, we can calculate $D^{\mathrm{d}}(v)$ and $D^{\mathrm{u}}(v)$ by noting $D^{\mathrm{d}}(v) = A(v) f_{\mathrm{th}}^{\mathrm{d}} - B(v)$ and $D^{\mathrm{u}}(v) = A(v)\left(1 - f_{\mathrm{th}}^{\mathrm{u}}\right) - B(v)$.

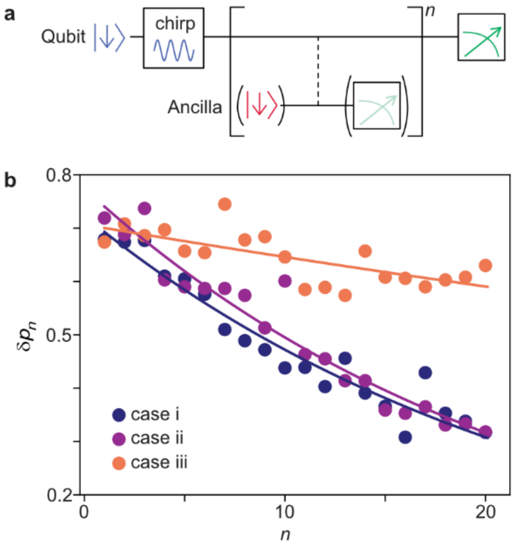

Supplementary Figure 1. Qubit spin decay in different conditions | a, Schematic for the control experiments. **b,** Qubit relaxation rates in different conditions. T_1^{tot} = 1.8, 1.7 and 8.4 ms for cases i, ii, iii, respectively.

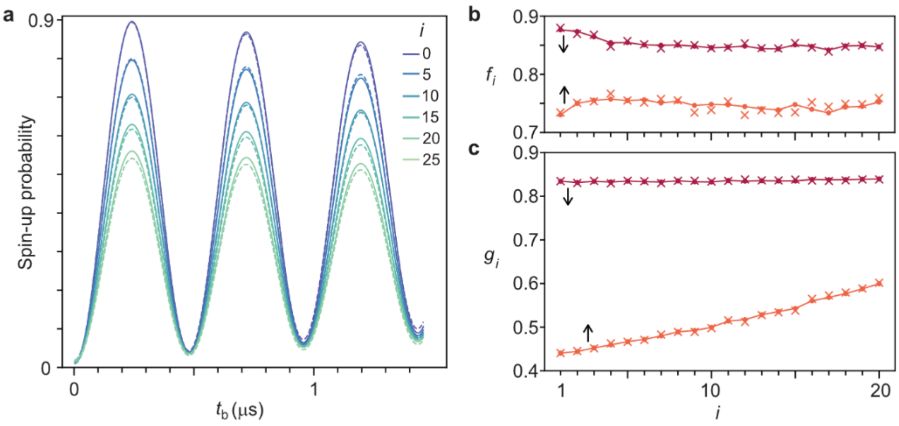

Supplementary Figure 2. Parameters obtained from the joint probability analysis | a, $p_i^{\uparrow} = 1 - p_i^{\downarrow}$ as a function of i. For brevity, we only show traces for i divisible by 5. Dashed and solid lines plot the results from the initial and later analysis steps, respectively. **b,** $f_i^{\downarrow(\uparrow)}$ as a function of i. Crosses and dots are the results from the initial and last analysis steps, respectively. **c,** $g_i^{\downarrow(\uparrow)}$ as a function of i. Crosses and dots are from the initial and last steps, respectively. Unlike $f_i^{\downarrow(\uparrow)}$ and $g_i^{\downarrow}$, $g_i^{\uparrow}$ shows a monotonic increase as a function of i, consistent with the qubit state relaxation from ↑ to ↓ between the i-th and 30th ancilla measurements.

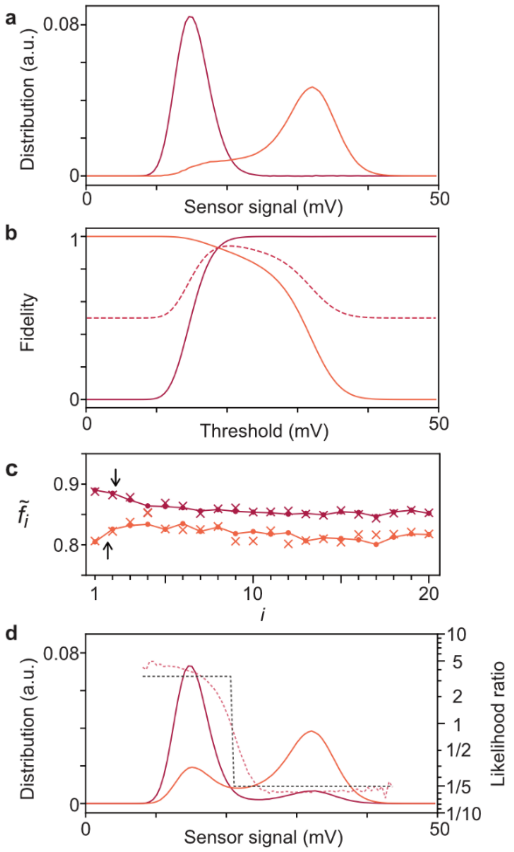

Supplementary Figure 3. Charge discrimination error | a, Sensor signal distributions (normalized histograms) for traces with blips (orange) and without blips (red). **b,** Charge discrimination fidelities for traces with and without blips (plotted in orange and red, respectively) as a function of the threshold value. The dashed line shows the average of these fidelities. **c,** The qubit-to-ancilla-blip conversion fidelities $\tilde{f}_i$ extracted from joint probability analysis in which the ancilla probability is obtained from the sensor signal distribution. $\tilde{f}_i^{\downarrow(\uparrow)}$ is plotted in red (orange). **d,** Sensor signal distributions for spin-down (red) and spin-up (orange) cases. Dotted lines show the ratio of these distributions (red) and the single-shot likelihood ratio for a given sensor signal used in the main text (black).
